# Impact of Preoperative Comorbidities on Hospital Stay in Patients Undergoing Hip Arthroplasty: A Retrospective Cohort Study

**DOI:** 10.7759/cureus.85301

**Published:** 2025-06-03

**Authors:** Andrei Danet, Razvan Spiridonica, Adrian Cursaru, Bogdan Cretu, Bogdan Serban, Mihai Aurel Costache, Catalin Cirstoiu

**Affiliations:** 1 Cardiac Surgery, Carol Davila University of Medicine and Pharmacy, Bucharest, ROU; 2 Cardiac Surgery, University Emergency Hospital Bucharest, Bucharest, ROU; 3 Orthopedics and Traumatology, University Emergency Hospital Bucharest, Bucharest, ROU; 4 Orthopedics and Traumatology, Carol Davila University of Medicine and Pharmacy, Bucharest, ROU; 5 Orthopaedics and Traumatology, Carol Davila University of Medicine and Pharmacy, Bucharest, ROU

**Keywords:** biochemical markers, coagulation, hematology, hip arthroplasty, postoperative complications, renal function, risk stratification

## Abstract

This retrospective study investigated the relationship between preoperative comorbidities and hospitalization duration in patients undergoing total hip arthroplasty. Conducted at a single tertiary center, the analysis included 85 patients, who were stratified based on length of hospital stay into two groups: short stay (≤10 days) and prolonged stay (>10 days). Comorbidities were recorded as binary variables, and statistical tests, including Chi-square analysis, were applied to identify associations with hospitalization length. The most prevalent condition was arterial hypertension, followed by congestive heart failure, hepatic steatosis, and atrial fibrillation. Significant associations were found between prolonged hospitalization and specific comorbidities, notably congestive heart failure, liver cirrhosis, mitral regurgitation, pleural effusion, and arterial hypertension. In contrast, other conditions such as diabetes mellitus and depression did not significantly affect length of stay. These findings emphasize the role of cardiovascular and hepatic comorbidities as key predictors of delayed recovery and support the integration of individualized risk assessment into perioperative planning to improve clinical outcomes and optimize resource utilization.

## Introduction

Total hip arthroplasty (THA) is a widely performed orthopedic procedure that plays a crucial role in alleviating pain, restoring joint function and improving the quality of life for patients suffering from advanced degenerative joint diseases and hip trauma [1]. As a highly effective surgical intervention, THA enables patients to regain mobility and resume daily activities, making it the preferred treatment for conditions such as osteoarthritis, avascular necrosis, and femoral fractures. However, despite its benefits, THA is associated with a range of postoperative complications, including hematological, biochemical, and coagulation abnormalities, all of which require careful perioperative management to ensure optimal patient outcomes [1,2].

The demand for THA has increased significantly worldwide, driven by the aging population and the rising incidence of degenerative joint diseases [3]. The elderly population is particularly vulnerable to complications following THA due to age-related comorbidities such as hypertension, diabetes, osteoporosis, and renal dysfunction. Proximal femur fractures, which are commonly observed in patients over 60 years of age, further contribute to the complexity of surgical management. These fractures, often resulting from low-energy trauma and bone fragility, are linked to high morbidity and mortality rates, emphasizing the importance of individualized risk assessment [4,5]. Moreover, metabolic disturbances such as electrolyte imbalances, postoperative anemia, and renal impairment are frequent concerns that can prolong hospitalization and complicate rehabilitation [1].

To address these challenges, a robust statistical analysis of perioperative risk factors is essential. By evaluating key hematological and biochemical parameters before and after surgery, clinicians can identify patterns associated with adverse outcomes and implement targeted interventions to mitigate risks [4]. Furthermore, technological advancements such as 3D printing in arthroplasty are paving the way for more precise implant positioning and improved surgical outcomes, further reinforcing the need for data-driven decision-making in orthopedic care [6].

This study aims to provide a detailed statistical assessment of postoperative complications in patients undergoing THA, focusing on variations in coagulation markers, renal function, electrolyte balance, and hospitalization duration. By identifying the most significant risk factors, this research seeks to enhance perioperative management strategies, reduce complication rates, and improve overall patient recovery following hip arthroplasty.

## Materials and methods

Study design and population

This retrospective observational study was conducted on a cohort of 85 patients who underwent THA at a single institution. Data collection included patient demographics, comorbidities, and hospitalization duration. Patients were included in the study if they had undergone primary THA and hemiarthroplasty, were 24 years or older, had complete preoperative and postoperative laboratory data available, and had no history of active systemic infection at the time of surgery. Patients included in the study underwent THA or hemiarthroplasty primarily for either osteoarthritis or femoral neck fractures; notably, the majority of those with prolonged hospital stays (>10 days) had sustained femoral neck fractures, suggesting a higher perioperative risk profile in this subgroup. 

Exclusion criteria were applied to patients with a history of previous hip arthroplasty in the same joint, severe preoperative organ failure (renal, hepatic, or cardiac) requiring immediate intensive care, malignancies with active systemic disease at the time of surgery, or missing or incomplete laboratory and clinical data.

The sample size was based on the total number of eligible patients who underwent THA during the study period at our institution and met the inclusion and exclusion criteria. This approach ensured that all available data were used, resulting in a cohort of 85 patients. While constrained by the retrospective design, this sample provided sufficient data to identify statistically significant associations between several comorbidities and hospitalization duration.

Data collection and variables

Patient data were extracted from electronic medical records and included demographic information, comorbidity profiles, and hospitalization duration. Comorbidities were coded based on preoperative diagnoses documented in the medical charts and included a wide spectrum of systemic conditions such as cardiovascular, hepatic, renal, and metabolic disorders, among others.

Hospitalization duration was analyzed by grouping patients into two categories: those with a short stay of 10 days or fewer and those with a prolonged stay exceeding 10 days. Each comorbidity was documented as a binary variable, indicating either its presence or absence in the patient's medical history. To better understand the distribution of these conditions within the cohort, descriptive statistics were calculated, including the mean, standard deviation, skewness, and kurtosis, which provided insight into the asymmetry and concentration of each variable's distribution.

Statistical analysis

Descriptive statistics, including mean, standard deviation, median, minimum, and maximum values, were used to analyze numerical variables. Skewness and kurtosis were calculated to assess data distribution. Categorical variables, such as comorbidities and prosthesis type, were expressed as frequencies and percentages. The association between specific preoperative comorbidities and hospitalization length was assessed using Chi-square tests, with statistical significance set at p<0.05. Pearson correlation analyses were used to explore relationships between continuous variables. Data visualization involved heatmaps and distribution plots to highlight patterns and group differences. Analyses were conducted using Python 3.9.13, and the library version used was Matplotlib 3.5.2.

Ethical considerations

All participants in the study gave informed consent for clinical follow-up with respect to ethical guidelines. Such consent ensures that the highest standards of ethical conduct are observed in relation to protecting the rights of patients and that the patient data obtained are maintained confidentially.

## Results

The patient cohort was stratified based on the duration of hospitalization following THA into two groups: those discharged in 10 days or fewer (short stay) and those requiring hospitalization for more than 10 days (prolonged stay). Out of the total number of patients, 59 (69.4%) experienced a short stay, while 26 (30.6%) had prolonged hospitalizations. This distribution reflects a relatively favorable recovery trajectory for the majority, yet highlights a substantial proportion of patients facing extended recovery periods (Table 1).

**Table 1:** Distribution of patients by hospitalization duration

Hospitalization duration	Patients, n (%)
Short (≤10 days)	59 (69.4%)
Prolonged (>10 days)	26 (30.6%)

To evaluate potential predictors of prolonged hospitalization, a broad range of comorbidities was assessed. Each condition was recorded as present or absent, and descriptive statistics were computed to explore the distribution characteristics within the patient population.

The patient cohort undergoing THA revealed a diverse and clinically complex background. Among the comorbidities analyzed, arterial hypertension stood out as the most prevalent, affecting 65 (76.5%) of the patients. This aligns with the well-established association between advanced age, cardiovascular risk, orthopedic surgical candidacy, and diabetes mellitus (11 (12.9%)), which are known to exacerbate surgical risk profiles and potentially prolong recovery. Less common, yet clinically significant, were pleural effusion (18 (21.2%)), atrial fibrillation (13 (15.3%)), and depression (11 (12.9%)), conditions that may not directly influence the surgical field but can critically alter perioperative stability and rehabilitation potential. Certain diagnoses, such as rheumatoid arthritis (1 (1.2%)), gastric ulcer (5 (5.9%)), and tuberculosis history (3 (3.5%)), were relatively rare in this population, but due to their systemic implications, they should not be dismissed in individualized risk assessments.

Interestingly, only 9 (10.6%) patients had a documented neoplastic history, reflecting the general contraindication of elective joint replacement in active malignancy cases. Hepatitis B infection and liver cirrhosis were found in just 2 (2.4%) and 3 (3.5%) of patients, respectively.

From a statistical perspective, high skewness and kurtosis values were observed across many conditions, indicating a heavily asymmetric distribution, where the vast majority of patients did not present the comorbidity, while a small subset carried a disproportionate burden of risk. This highlights the importance of tailoring perioperative protocols to this high-risk minority (Table 2).

**Table 2:** Prevalence of comorbidities with descriptive statistics *Skewness; **kurtosis

Comorbidity	Yes, n (%)	No, n (%)	Mean	SD	S*	K**
Asthma	8 (9.4%)	77 (90.6%)	0.094	0.294	2.83	6.155
Arterial hypertension	65 (76.5%)	20 (23.5%)	0.765	0.427	-1.271	-0.395
Acute renal failure	0 (0.0%)	85 (100.0%)	0	0	0	0
Chronic kidney disease	7 (8.2%)	78 (91.8%)	0.082	0.277	3.093	7.751
Hepatic steatosis	19 (22.4%)	66 (77.6%)	0.224	0.419	1.351	-0.179
Diabetes mellitus	11 (12.9%)	74 (87.1%)	0.129	0.338	2.248	3.127
Pleural effusion	18 (21.2%)	67 (78.8%)	0.212	0.411	1.436	0.064
Neoplasm (cancer)	9 (10.6%)	76 (89.4%)	0.106	0.31	2.608	4.917
Depression	11 (12.9%)	74 (87.1%)	0.129	0.338	2.248	3.127
Atrial fibrillation	13 (15.3%)	72 (84.7%)	0.153	0.362	1.963	1.899
Mitral regurgitation	5 (5.9%)	80 (94.1%)	0.059	0.237	3.818	12.877
Tricuspid regurgitation	2 (2.4%)	83 (97.6%)	0.024	0.152	6.4	39.903
Pulmonary hypertension	8 (9.4%)	77 (90.6%)	0.094	0.294	2.83	6.155
Congestive heart failure	28 (32.9%)	57 (67.1%)	0.329	0.473	0.739	-1.489
Hepatitis B infection	2 (2.4%)	83 (97.6%)	0.024	0.152	6.4	39.903
Rheumatoid arthritis	1 (1.2%)	84 (98.8%)	0.012	0.108	9.22	85
Liver cirrhosis	3 (3.5%)	82 (96.5%)	0.035	0.186	5.128	24.879
Gastric ulcer	5 (5.9%)	80 (94.1%)	0.059	0.237	3.818	12.877
Tuberculosis history	3 (3.5%)	82 (96.5%)	0.035	0.186	5.128	24.879

This binary heatmap illustrates the association between comorbid conditions and hospitalization duration, classified as either short (≤10 days) or prolonged (>10 days). The color gradient reveals how the presence of each comorbidity aligns with hospital stay length.

**Figure 1:**
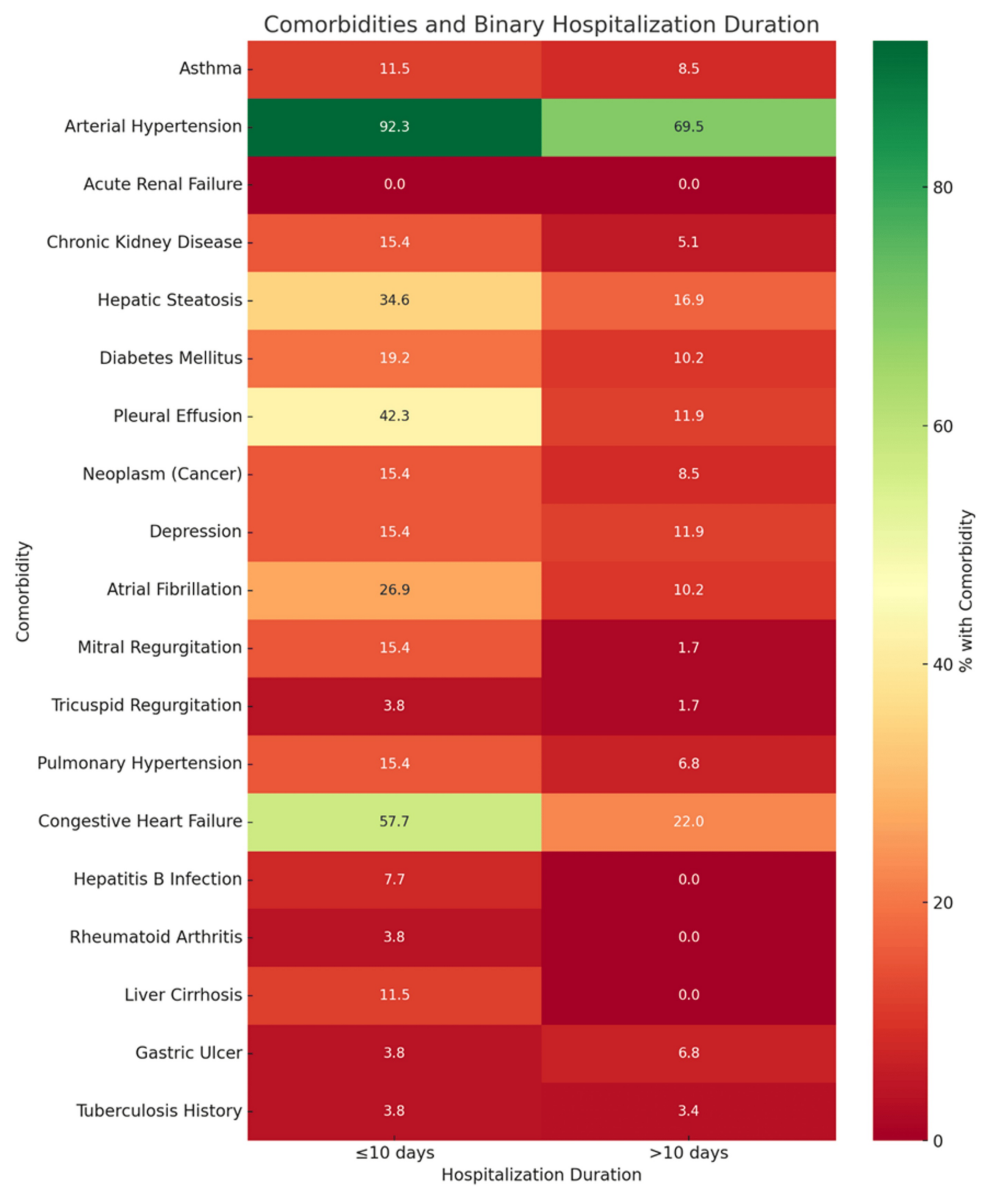
Binary heatmap showing comorbidity distribution by hospitalization duration

The heatmap revealed that most comorbidities, including arterial hypertension, congestive heart failure, atrial fibrillation, and pleural effusion, were more frequently observed among patients with shorter hospitalization durations. This suggests that the presence of these conditions did not necessarily lead to extended inpatient recovery in this cohort. Interestingly, no comorbidity was predominantly concentrated in the prolonged stay group, indicating that factors beyond the presence of a single comorbidity, such as combined disease burden, perioperative complications, or social factors, may play a more decisive role in prolonging hospitalization following THA (Figure 1).

**Figure 2:**
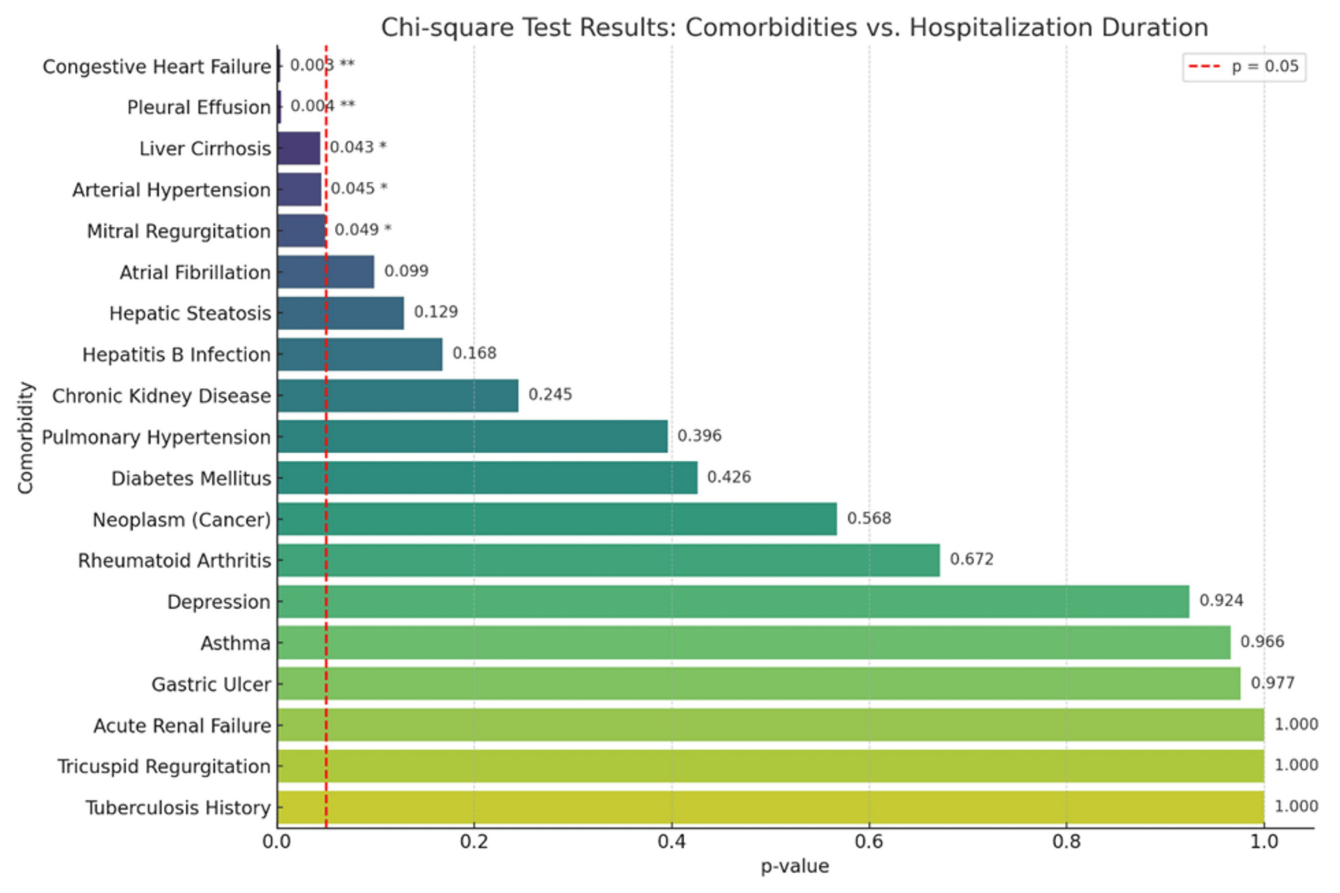
Bar chart of comorbidities with Chi-square p-values *p-value <0.05; **p-value <0.01

To quantify the strength of association between individual comorbidities and length of hospital stay, Chi-square tests were performed. Five comorbidities demonstrated statistically significant relationships with hospitalization duration. The chart illustrates the strength of association between individual comorbidities and hospitalization duration, measured through Chi-square tests. Each bar represents a specific condition, with shorter bars and lower p-values indicating a stronger statistical relationship. A red dashed line marks the threshold of statistical significance at p=0.05 (Figure 2). Conditions that fall to the left of this line are considered significantly associated with how long patients remained hospitalized. Among the analyzed comorbidities, five showed statistically significant differences. Congestive heart failure was much more common in patients who stayed longer than 10 days in the hospital, highlighting its role as a major factor in recovery and discharge timing. Pleural effusion followed a similar pattern, appearing far more frequently in the prolonged stay group. Liver cirrhosis was found exclusively in patients with extended hospitalization, suggesting it may pose barriers to rapid recovery. Arterial hypertension, while common overall, was disproportionately higher in those hospitalized for more than 10 days. Mitral regurgitation also leaned toward the prolonged stay group, appearing rarely in patients discharged earlier. These findings emphasize that cardiovascular and hepatic conditions are strongly linked to delayed postoperative recovery in this patient population (Table 3).

**Table 3:** Chi-square test results

Comorbidity	Short stay (<10 d), n	Prolonged stay (>10 d), n	Total, n	p-value	Chi-square value
Congestive heart failure	25	3	28	0.003	8.94
Pleural effusion	17	1	18	0.004	8.19
Liver cirrhosis	3	0	3	0.043	4.09
Arterial hypertension	49	16	65	0.045	4.01
Mitral regurgitation	4	1	5	0.049	3.87
Atrial fibrillation	10	3	13	0.099	2.71
Hepatic steatosis	13	6	19	0.129	2.31
Hepatitis B infection	1	1	2	0.168	1.9
Chronic kidney disease	5	2	7	0.245	1.35
Pulmonary hypertension	5	3	8	0.396	0.72
Diabetes mellitus	7	4	11	0.426	0.63
Neoplasm (cancer)	6	3	9	0.568	0.33
Rheumatoid arthritis	1	0	1	0.672	0.18
Depression	8	3	11	0.924	0.01
Asthma	5	3	8	0.966	0.01
Gastric ulcer	4	1	5	0.977	0
Acute renal failure	0	0	0	1	0
Tricuspid regurgitation	2	0	2	1	0
Tuberculosis history	2	1	3	1	0

## Discussion

Our study evaluated the relationship between pre-existing comorbidities and the duration of hospitalization in patients undergoing THA. The majority of patients (69.4%) were discharged within 10 days, while 30.6% experienced prolonged hospitalization. Among the comorbidities assessed, arterial hypertension emerged as the most prevalent condition, affecting over three-quarters of the cohort, followed by congestive heart failure, hepatic steatosis, and atrial fibrillation. Statistical analysis revealed that several comorbidities, particularly congestive heart failure, liver cirrhosis, pleural effusion, arterial hypertension, and mitral regurgitation, were significantly associated with extended hospital stays. These findings were supported by both visual heatmap analysis and Chi-square testing, underscoring the critical role of cardiovascular and hepatic comorbidities in predicting postoperative recovery trajectories. Conversely, other conditions such as diabetes mellitus and depression did not demonstrate a significant impact on length of stay (LOS), suggesting a more nuanced role in perioperative management. It is important to note that pleural effusion, as recorded in this study, may reflect a range of underlying conditions. While most cases were associated with cardiac comorbidities such as heart failure or valvular disease, the possibility of malignant or infectious causes cannot be excluded due to the retrospective nature of data collection. This heterogeneity limits interpretation and should be addressed in future studies with more detailed clinical and radiologic correlation. Overall, our results highlight the need for individualized perioperative strategies tailored to patients’ specific comorbidity profiles to improve recovery outcomes following hip arthroplasty. 

Our findings are consistent with those reported by another recent study, which analyzed data from the National Inpatient Sample and found significantly higher rates of complications, particularly urinary, cardiac, and pulmonary events, in patients with chronic kidney disease (CKD) or end-stage renal disease undergoing THA compared to those with osteoarthritis alone. Their study highlighted a progressive increase in complication rates from CKD stage 3 onward. Similarly, in our cohort, patients with renal dysfunction exhibited more frequent electrolyte disturbances, elevated creatinine, and longer hospital stays. While they provide broad, stage-based national insights, our single-center analysis adds clinical detail through perioperative laboratory parameters, reinforcing the need to incorporate renal status into preoperative risk assessment and care planning [7].

Angerame et al. [8] recently examined the necessity of routine perioperative laboratory tests in total hip and knee arthroplasty, concluding that extensive lab monitoring may not be required for all patients. Their study, which included 967 patients, found that only a small proportion of preoperative and postoperative tests led to actionable clinical interventions, particularly in relation to coagulation and metabolic panels. These results align with our findings, where postoperative coagulation abnormalities were observed in 28.2% of patients, with international normalized ratio (INR) levels exceeding 2, and 9.4% presenting with INR values above 4. This suggests that while some patients require close monitoring, routine testing for all may not be necessary.

Additionally, Angerame et al. [8] identified renal disease and diabetes as key predictors of postoperative laboratory abnormalities, particularly in complete metabolic panel results. Our study supports these findings, revealing that postoperative creatinine levels exceeded 2 mg/dL in 41.2% of patients, with 10.6% developing acute renal failure. This further highlights the importance of individualized perioperative risk assessment, focusing laboratory evaluations on high-risk patients instead of applying a one-size-fits-all approach.

Moreover, their study emphasized the economic burden of unnecessary laboratory testing in arthroplasty patients and advocated for a more selective testing approach to improve efficiency while maintaining patient safety [8]. Our findings reinforce this perspective, as we observed significant biochemical and hematological variations that, while clinically relevant, suggest the need for a more targeted testing strategy rather than universal postoperative monitoring. Together, these studies support the shift toward a patient-specific approach in perioperative laboratory assessments, balancing optimized clinical outcomes with responsible healthcare resource utilization.

Curlewis et al. [9] reviewed systemic complications following joint arthroplasty and found them to be more common than regional ones, with venous thromboembolism, pneumonia, and acute kidney injury being the most frequent. They identified preoperative comorbidities, particularly renal dysfunction, diabetes, and elevated American Society of Anesthesiologists (ASA) scores, as key predictors of complications and mortality [9]. These findings align with our results, where conditions such as CKD and diabetes were associated with longer hospital stays and abnormal perioperative lab values. While their review covered multiple joint types, our study offers a focused analysis of hip arthroplasty patients, reinforcing the importance of individualized risk assessment based on comorbidity profiles.

A recent study investigated perioperative complications in patients aged ≥80 years undergoing hip and knee revision arthroplasty and found that, despite a higher prevalence of systemic complications such as anemia, delirium, and cardiovascular events, advanced age was not associated with increased length of hospital stay or readmission rates. These findings partially align with our results, where older patients also exhibited increased rates of anemia and comorbidities such as hypertension and cardiovascular disease [10]. However, in contrast to their conclusions, our study found that these factors contributed to extended hospitalization in certain cases, suggesting that age-related vulnerability may be more pronounced in primary arthroplasty settings when compounded by multiple biochemical and systemic risk markers.

Another study analyzed 1,082 patients undergoing total knee arthroplasty (TKA) and identified age, female sex, and comorbidities, especially heart disease, as key predictors of prolonged hospital stay, while caregiver support and higher BMI were associated with shorter LOS. Although their study focused on knee arthroplasty, these findings align with our observations in hip arthroplasty, where older age and comorbidities such as hypertension and CKD were similarly linked to extended hospitalization [11]. Additionally, both studies emphasize the relevance of individualized preoperative planning to optimize LOS and resource allocation, reinforcing the broader role of biopsychosocial factors in postoperative recovery.

Postoperative anemia was also a significant concern, with 21.2% of patients exhibiting hemoglobin levels below 10 g/dL and 8.2% suffering from severe anemia (hemoglobin or Hb <8 g/dL). Given the association between anemia and delayed recovery, longer hospital stays, and increased morbidity, optimizing preoperative hemoglobin levels and considering blood conservation strategies may improve postoperative outcomes. Recent studies have challenged the necessity of routine postoperative hematological monitoring in THA, citing a low incidence of clinically significant anemia. Howgate et al. [12] found that only 0.8% of 367 patients undergoing elective THA and TKA required a transfusion, despite routine Hb and hematocrit testing in 67% of cases​. Similarly, our study supports a selective approach, as while 21.2% of patients experienced postoperative anemia, only 8.2% had severe cases (Hb <8 g/dL), emphasizing the need for risk-based monitoring.

Howgate et al. [12] also identified preoperative hemoglobin <12.5 g/dL and hematocrit <40% as key predictors of transfusion, reinforcing the importance of preoperative optimization, which our findings support. Additionally, they highlighted the financial burden of unnecessary lab testing, estimating a potential annual cost saving. Our results align with this perspective, suggesting that targeted postoperative testing for high-risk patients can improve resource utilization without compromising patient safety, further advocating for personalized perioperative management over routine testing.

In a recent meta-analysis by Zhang et al. [13], the impact of preoperative anemia on postoperative outcomes in total joint arthroplasty was extensively analyzed. The study found that preoperative anemia was present in 22% of patients undergoing total hip and knee arthroplasty and was associated with increased postoperative complications, longer hospital stays, higher transfusion rates, and greater mortality risk​. These findings align with our study, which also observed significant postoperative alterations in hematological parameters, particularly hemoglobin levels, leading to a high prevalence of postoperative anemia. In our cohort, 21.2% of patients had hemoglobin levels below 10 g/dL, with 8.2% presenting severe anemia (<8 g/dL). The meta-analysis further highlighted that anemic patients had a substantially higher risk of deep vein thrombosis and infections, which were also prevalent concerns in our study [13]. Our results corroborate these findings, as postoperative coagulation disturbances were evident, with INR levels rising above 2 in 28.2% of patients and exceeding 4 in 9.4%, indicating an increased risk of bleeding and thrombotic events.

To further contextualize our findings, Table 4 presents a summary of recent studies addressing perioperative outcomes, comorbidity impact, and predictors of hospitalization duration in patients undergoing joint arthroplasty.

**Table 4:** Summary of key literature supporting the discussion CHF, congestive heart failure; CKD, chronic kidney disease; DVT, deep vein thrombosis; ESRD, end-stage renal disease; LOS, length of stay; THA, total hip arthroplasty; TKA, total knee arthroplasty

Study	Citation	Study focus	Main findings	Relevance to the present study
Fox et al., 2023	[7]	Complications in THA patients with CKD/ESRD	ESRD significantly increases risks after THA, including pulmonary and cardiac complications	Reinforces CKD as a critical predictor of poor postoperative outcomes
Angerame et al., 2021	[8]	Value of routine perioperative labs	Most labs non-actionable; comorbid patients benefit most from targeted testing	Supports selective lab testing in patients with risks (e.g., CKD, CHF)
Curlewis et al., 2023	[9]	Systemic complications in arthroplasty	Systemic (esp. cardiac) complications are more common than local; comorbidities drive outcomes	Supports holistic perioperative evaluation
Di Matteo et al., 2023	[10]	Complications in elderly THA/TKA patients	Elderly with optimized perioperative care have acceptable complication profiles	Adds nuance to age-related risk in elderly patients
Tornese et al., 2024	[11]	LOS predictors after TKA	Age, comorbidities, and female sex extend hospital stay; caregiver presence reduces it	Aligns with demographic and clinical predictors in your study
Howgate et al., 2024	[12]	Utility of routine postoperative H&H tests	Most H&H tests unnecessary; pre-op Hgb and Hct best predict transfusion need	Supports a selective lab monitoring strategy
Zhang et al., 2024	[13]	Meta-analysis: Preoperative anemia in THA/TKA	Anemia linked to ↑ transfusions, infections, DVT, readmission, mortality, and LOS	Strongly supports your findings, linking hemoglobin levels to worse outcomes

Given that several comorbidities were significantly associated with extended hospitalization, these results reinforce the importance of individualized perioperative care protocols. Implementing stratified care pathways based on comorbidity profiles, especially for patients with cardiovascular or hepatic impairment, may improve outcomes, reduce hospitalization time, and optimize healthcare resource utilization.

This retrospective, single-center study has limited generalizability, and the small sample size may have reduced the ability to detect associations for less common comorbidities. The lack of multivariate analysis prevents adjustment for potential confounders such as age, BMI, or surgical approach, meaning observed associations should be interpreted as correlations rather than causative links. Standardized comorbidity indices such as the Charlson or ASA score were not used, limiting comparability with other studies. Additionally, outcomes were assessed only during hospitalization, without post-discharge follow-up or evaluation of long-term complications. The cost-effectiveness of targeted versus routine laboratory testing was also not explored. Despite these limitations, the findings highlight relevant trends and underscore the impact of cardiovascular and hepatic comorbidities on recovery following hip arthroplasty. Further multicenter prospective studies are needed to confirm and expand upon these results.

## Conclusions

This study highlights the significant influence of preoperative comorbidities on hospitalization duration in patients undergoing THA. Cardiovascular and hepatic conditions, particularly congestive heart failure, arterial hypertension, mitral regurgitation, pleural effusion, and liver cirrhosis, were strongly associated with prolonged hospital stays. These findings underscore the importance of integrating comorbidity screening into preoperative planning to better anticipate postoperative recovery needs. A targeted, patient-specific approach to perioperative management may improve outcomes, reduce hospitalization time, and optimize healthcare resource utilization. Further prospective studies with larger cohorts and multivariate analysis are warranted to validate these associations and support risk-adapted clinical pathways in orthopedic surgery.
